# Hitting the hidden: arming the immune system for the next zoonotic coronavirus spillover

**DOI:** 10.1172/JCI203781

**Published:** 2026-03-16

**Authors:** Charlie Fricke, Stanley Perlman

**Affiliations:** Department of Microbiology and Immunology, University of Iowa, Iowa City, Iowa, USA.

## Abstract

Coronaviruses, both known and yet to emerge, pose persistent zoonotic and pandemic threats. While current parenteral COVID-19 mRNA vaccines effectively mitigate severe disease caused by SARS-CoV-2, they primarily elicit systemic immunity restricted to specific variants within clade 1b of the sarbecovirus subgenus and provide limited mucosal protection. Addressing these shortcomings, Cheang et al. developed a DC-targeting intranasal booster vaccine that induces robust and durable mucosal and systemic immunity across sarbecovirus clades 1a and 1b. This study highlights a promising strategy for pan-sarbecovirus vaccines by leveraging mucosal immune induction to prevent viral transmission and enhance pandemic preparedness.

## Redefining boosters for broad and durable coronavirus immunity

The introduction of mRNA-based platforms into clinical practice during the SARS-CoV-2 pandemic revolutionized vaccine development by providing an unprecedentedly rapid method to generate vaccines tailored to emerging viral variants (1). Despite this advancement, viral evolution continues to outpace new vaccine formulations, leading to the frequent emergence of immune-evasive variants (2). This dynamic has necessitated the implementation of seasonal booster doses, which, although effective, encountered declining public acceptance over time (3). The development of a universal vaccine that provides durable, cross-variant protection could not only reduce the need for frequent updates but also enhance public compliance and global vaccination rates (4).

**Figure 1:**
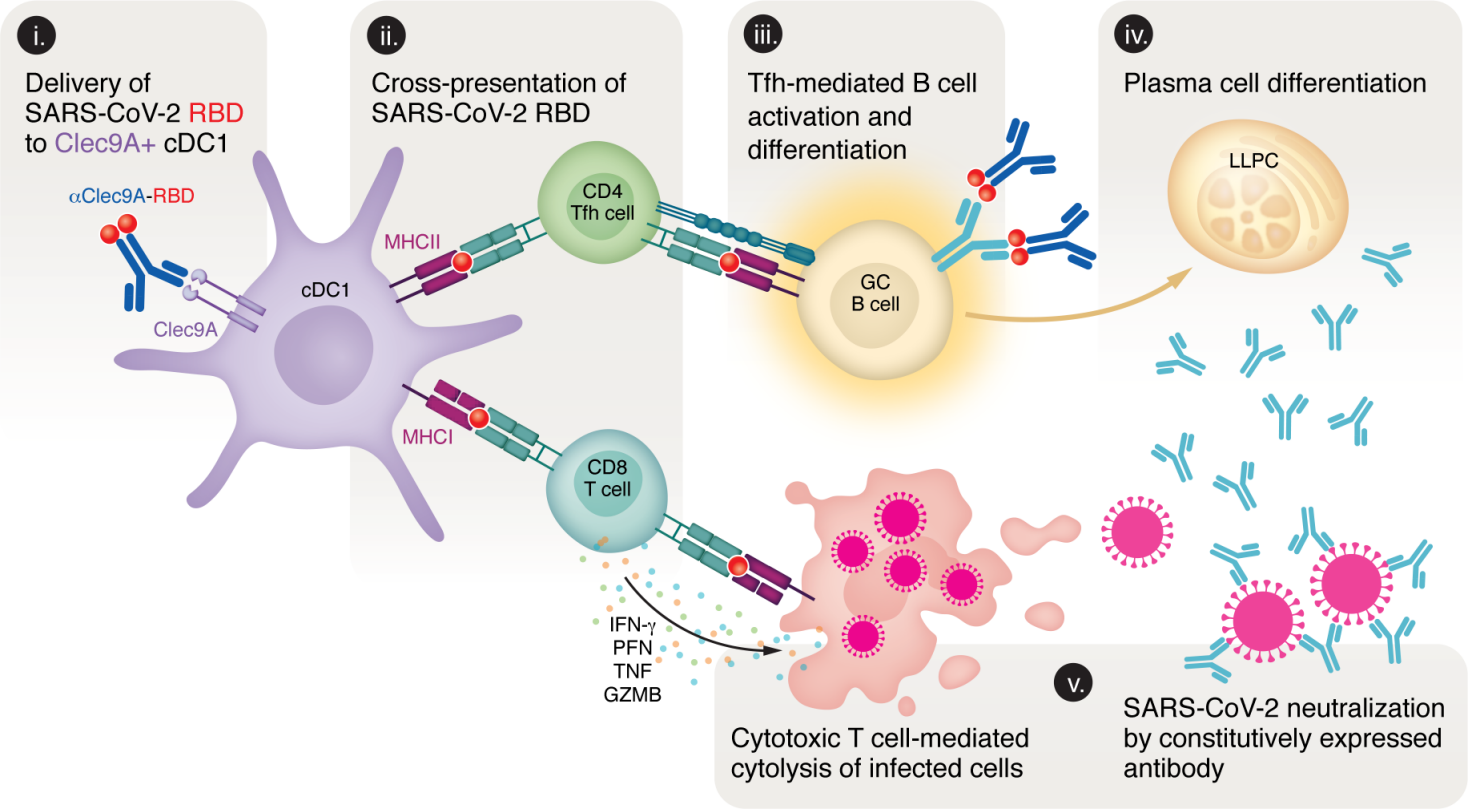
Mechanism of Clec9A-targeted vaccine–induced immunity. The bivalent mucosal booster vaccine developed by Cheang et al. utilized Clec9A-targeted delivery of SARS-CoV-2 RBD to cDC1s, enabling humoral and cellular immune activation (5). (i) The αClec9A–RBD fusion antibody binds Clec9A on cDC1s, facilitating efficient antigen uptake and processing. (ii) Processed antigens are cross-presented via MHC class I (MHCI) to CD8^+^ T cells and presented via MHC class II (MHCII) to CD4^+^ T cells, inducing cytotoxic and helper functions, respectively. (iii) Tfh–B cell interactions promote GC B cell activation and affinity maturation. (iv) GC B cells differentiate into plasma cells that secrete high-affinity neutralizing antibodies. (v) These antibodies neutralize SARS-CoV-2, while CD8^+^ T cell–mediated cytolysis contributes to durable, cross-protective immunity.

Cheang et al. addressed this challenge by developing a bivalent mucosal booster vaccine (Clec9A^OMNI^) that combines receptor-binding domains (RBDs) from SARS-CoV-1 (clade 1a) and SARS-CoV-2 variant Omicron XBB.1.5 (clade 1b) (5). Mechanistically, the constructs use a Clec9A-targeting monoclonal antibody to deliver the antigens directly to conventional type 1 DCs (cDC1), which are highly efficient in priming CD4^+^ and CD8^+^ T cell responses and sustaining germinal center (GC) reactions (Figure 1) (6, 7). To assess the efficacy of their vaccine, the authors utilized a prime-boost mouse model. Animals were first immunized with 2 doses of the Pfizer-BioNTech Comirnaty mRNA vaccine, since most humans have been vaccinated with this or a similar vaccine (8). Three months later, they received a booster immunization with either the bivalent BA.4/5 Comirnaty mRNA vaccine or Clec9A-based constructs. Cheang et al. first examined the effects of systemic, subcutaneous delivery of Clec9A^XBB^ (carrying Omicron XBB.1.5 RBD) and Clec9A^CoV1^ (carrying SARS-CoV-1 RBD), which revealed complementary properties: Clec9A^XBB^ elicited sustained but clade 1b–restricted neutralizing antibodies (nAbs), whereas Clec9A^CoV1^ conferred broader cross-clade (1a and 1b) neutralization that declined more rapidly. Combining both constructs into a single formulation (Clec9A^OMNI^) overcame these limitations, eliciting durable cross-clade humoral and cellular immunity that outperformed the bivalent Comirnaty mRNA booster, with higher and longer-lasting nAb titers, stronger GC activity, and sustained lung protection against SARS-CoV-2 BA.1 challenge for up to 6 months (5).

To determine whether mucosal delivery could further enhance protection, the authors administered Clec9A^OMNI^ intranasally, resulting in potent and persistent antibody and T cell responses in both systemic and respiratory compartments (5). In subsequent viral challenge experiments with the Omicron BA.1 variant or mouse-adapted SARS-CoV-2, Clec9A^OMNI^ conferred near-complete protection in both the upper and lower airways for up to 6 months after boost, in contrast to waning efficacy observed after bivalent mRNA boosting (5). Together, these results highlight Clec9A^OMNI^ as a rationally engineered booster that induces broad, durable, systemic, and mucosal immunity and outperforms current mRNA vaccines in a preclinical mouse model.

## Antigenic diversity broadens and sustains immunity

The findings reported by Cheang et al. further illustrate how antigenic diversity fundamentally reshapes the outcome of immune responses (5). Sequential or homologous boosting with ancestral or Omicron-based mRNA vaccines could risk reinforcing clade-specific memory, a phenomenon known as immune imprinting, thereby limiting the breadth of the immune response (9–11). In contrast, incorporating divergent RBDs from distinct sarbecovirus clades (1a and 1b) recruits previously subdominant cross-reactive B and T cell clones, expanding the range of recognized epitopes and broadening the nAb response (5). Notably, intranasal delivery of Clec9A^OMNI^ induced a population of triple cross-reactive B cells recognizing the RBDs of ancestral SARS-CoV-2, XBB.1.5, and SARS-CoV-1 across nasal-associated lymphoid tissue, lung, and spleen, which was not observed after conventional SARS-CoV-2 Omicron-based mRNA boosting. These B cells gave rise to broadly neutralizing antibodies that persisted for at least 6 months, supported by sustained GC activity and the presence of RBD-specific T follicular helper (Tfh) cells.

The bivalent antigenic exposure also fostered balanced CD4^+^ and, to a lesser extent, CD8^+^ T cell responses. This response combined de novo priming of clade 1a–specific T cells with the recall of cross-reactive memory responses from prior SARS-CoV-2 RBD exposure (5). Beyond SARS-CoV-2 and SARS-CoV-1, Clec9A-based vaccination elicited T cell responses against sarbecoviruses from both clades 1a and 1b, including bat coronaviruses WIV1 and RaTG13 and pangolin coronavirus GX-P5L (5). Antibody profiling confirmed broad RBD recognition and neutralization across this phylogenetic spectrum (5), underscoring the capacity to target conserved epitopes shared by multiple coronaviruses with zoonotic potential. These findings establish antigenic diversity, combined with targeted antigen delivery to DCs, as a powerful driver of immune breadth, positioning antigen diversification as a guiding principle for next-generation vaccine design.

Importantly, antigenic variation not only broadened specificity but also enhanced immune durability. Cheang et al. demonstrated that, unlike the bivalent Omicron-based mRNA vaccine, which failed to establish a stable long-lived plasma cell (LLPC) compartment in the bone marrow and exhibited rapid antibody waning, mucosal Clec9A-based boosters sustained robust GC responses and promoted the differentiation of RBD-specific B cells into LLPCs capable of continuous antibody secretion. These durable humoral responses persisted for at least 6 months after boost and correlated with the persistence of GC B cells and Tfh cells, indicating ongoing immune maturation (5). Collectively, these findings demonstrate that antigenic diversity enhances both the breadth of immune recognition and the longevity of protective immunity against sarbecoviruses.

## Mucosal immunity as a cornerstone of future vaccines

Despite their ability to generate systemic immune responses, current mRNA-based COVID-19 vaccines fail to elicit robust immune responses in the respiratory mucosa, the initial site of SARS-CoV-2 entry (12, 13). This limitation underscores the ongoing need for vaccine approaches that can induce both systemic and mucosal immunity. While parenteral mRNA vaccines prevent severe disease, they permit viral replication and transmission in the upper airways, allowing breakthrough infection and viral shedding among vaccinated individuals (12, 14). Cheang et al. show that nasal administration of the dual-antigen Clec9A^OMNI^ construct induced strong mucosal and systemic antibody responses, including secretory IgA and IgG in the bronchoalveolar and nasal lavage fluids (5). Their vaccine also promoted the generation of tissue-resident memory T cells and RBD-specific LLPCs within the respiratory tract (5), immune populations less robustly established by intramuscular mRNA vaccination (15, 16). In viral challenge experiments, nasally boosted animals demonstrated near-complete protection in both upper and lower respiratory tracts, with viral titers remaining at or below detection levels up to 6 months after boost. In contrast, parenterally mRNA-boosted animals displayed breakthrough infection and residual viral replication. The persistence of mucosal antibodies and memory cell subsets correlated with sustained viral control (5), highlighting the crucial role of local immunity in preventing reinfection and transmission.

Although the translation of nasal vaccination to human use will require further optimization, recent advances in mucosal delivery and adjuvant engineering provide a strong foundation for progress (17). Mucosal tissues present a uniquely challenging environment for vaccination, since they are continuously exposed to diverse microorganisms and protected by chemical and physical barriers (18). Consequently, mucosally delivered vaccines often fail to cross these barriers and thus do not generate protective immunity (17). Cheang et al. overcame this limitation using a DC-targeting Clec9A antibody strategy, which enabled efficient antigen delivery and direct immune activation within the respiratory mucosa. They used polyinosinic:polycytidylic acid (poly I:C) to potentiate mucosal immune activation (5). Poly I:C and its stabilized derivative have undergone limited evaluation in early-phase human cancer vaccine trials (19), but no vaccine containing poly I:C or its analogs is currently licensed for human use to protect from viral diseases. Moving forward, research should prioritize the development of safe mucosal adjuvants and innovative delivery systems that protect antigen integrity and enhance mucosal immune responses. Additionally, mucosal vaccination offers practical advantages. Needle-free delivery simplifies administration, enhances accessibility, and may improve acceptance (e.g., in pediatric immunization). Together, these insights establish mucosal vaccination not merely as a complementary approach but as a central element in future coronavirus vaccine strategies for preventing infection, controlling transmission, and strengthening pandemic preparedness.

## Building the foundations for pan-coronavirus protection

The dual-clade, intranasal Clec9A^OMNI^ booster represents a new generation of vaccine design that integrates 3 hallmarks of effective global health security: antigenic breadth, mucosal targeting, and immune durability. By boosting systemic and inducing local immune activation, it achieved near-sterilizing protection across respiratory compartments, a level of defense that current intramuscular vaccines can not provide (5). The booster’s ability to elicit cross-clade neutralization and tissue-resident immunity suggests that broad protection against sarbecoviruses is achievable. From a translational perspective, the Clec9A antibody platform functions as a modular plug-and-go system that can be rapidly adapted to emerging sarbecoviruses using existing monoclonal antibody production infrastructure. Future work should focus on translating these preclinical findings into human trials, refining delivery methods, and more thoroughly identifying correlates of mucosal protection (e.g., IgA persistence) (20). By broadening immune recognition and mitigating imprinting effects, this strategy strengthens preparedness against potential zoonotic spillovers from bats and other animal reservoirs, the hidden threats that seed future pandemics (21).

This approach exemplifies a principle of hitting the hidden: proactively targeting the immunological blind spots that enable new coronaviruses to emerge and spread. Ultimately, Cheang et al. provide a compelling framework for next-generation coronavirus vaccines that shift focus from chasing variants to proactive immune protection, transforming pandemic response from containment to true prevention.

## Funding support

This work is the result of NIH funding, in whole or in part, and is subject to the NIH Public Access Policy. Through acceptance of this federal funding, the NIH has been given a right to make the work publicly available in PubMed Central.

- NIH grant R01 AI129269.
- Aegis COVID-19 study, administered through the Indiana University (IU) School of Public Health-Bloomington and supported by multiple anonymous donations to the IU Foundation and by Eli Lilly and Company.

## Version 1 03/16/2026

Electronic publication
